# Risk-Aware Reinforcement Learning with Dynamic Safety Filter for Collision Risk Mitigation in Mobile Robot Navigation

**DOI:** 10.3390/s25175488

**Published:** 2025-09-03

**Authors:** Bingbing Guo, Guina Wang, Yiyang Chen, Yue Gao, Qian Xie

**Affiliations:** 1 School of Mechanical and Electrical Engineering, Soochow University, Suzhou 215137, China; 20234229033@stu.suda.edu.cn; 2 Jiangsu Eazytec Co., Ltd., Wuxi 214205, China; xieqian@eazytec.com

**Keywords:** robot navigation, obstacle avoidance, safety filter, reinforcement learning

## Abstract

Mobile robots face collision risk avoidance challenges in dynamic environments, necessitating that we address the safety and adaptability shortcomings of traditional navigation methods. Traditional methods rely on predefined rules, making it difficult to achieve flexible, safe, and real-time obstacle avoidance in complex, dynamic environments. To address this issue, a risk-aware, dynamic, adaptive regulation barrier policy optimization (RADAR-BPO) method is proposed, combining proximal policy optimization (PPO) with the control barrier function (CBF). RADAR-BPO generates exploratory actions using PPO and constructs a real-time safety filter using the CBF. This method uses quadratic programming to minimize risky actions, thereby ensuring safe obstacle avoidance while maintaining navigation efficiency. Testing of three phased learning environments in the ROS Gazebo simulation environment demonstrated that the proposed method achieves an obstacle avoidance success rate of nearly 90% in complex, dynamic, multi-obstacle environments and improves the overall mission success rate, validating its robustness and effectiveness in complex dynamic scenarios.

## 1. Introduction

Currently, the convergence of artificial intelligence (AI), the Internet of Things (IoT), and automation technologies is facilitating intelligent transformation across various industries [1,2,3]. Robotic technology represents a key driver of this evolution [4]. Advancements in intelligent algorithms and hardware enable robots to undertake increasingly complex tasks. As a critical subset of robots, mobile robots are being deployed extensively in industry [5,6], logistics [7], and agriculture [8].

Despite their diverse designs and functionalities, mobile robots commonly face challenges in safe obstacle avoidance and efficient navigation during operation [9]. In dynamic and complex environments, they must accurately identify obstacles, plan safe trajectories, and make real-time adjustments [10]. Ensuring task completion necessitates advanced sensor systems for real-time environmental perception [11]. Moreover, using intelligent algorithms to process information, execute dynamic path planning, and adapt to uncertainties is critical, and enhancing these capabilities is key to ensuring efficient and safe operation in complex environments [12,13,14].

Beyond traditional obstacle avoidance, navigating shared environments requires socially aware navigation capabilities [15]. Robots must not only avoid collisions but also adhere to social norms, exhibit predictable behavior, and respect personal space to achieve smooth and comfortable human–robot coexistence [16,17]. This involves understanding and predicting the motivations and intentions of human behavior and has become a research focus in the field of human–robot interaction (HRI) [18,19]. However, although the ultimate goal of social navigation is to achieve natural and comfortable human–machine coexistence, collision risk mitigation remains the most basic and critical safety prerequisite. Any social navigation strategy must be based on absolute collision avoidance in order to further optimize comfort and efficiency [20,21].

While techniques have previously been developed for obstacle avoidance and navigation by mobile robots, significant limitations persist [22,23]. Traditional control methods such as the artificial potential field (APF) method [24] and dynamic window approach (DWA) [25], often reliant on predefined rules and fixed path planning, perform adequately in simple tasks but lack adaptability and flexibility in complex, dynamic environments [26,27,28]. Their limited capacity for autonomous adjustment frequently results in decision errors or inefficiency when handling intricate scenarios [29]. Furthermore, the safety assurance mechanisms of conventional obstacle avoidance strategies remain inadequate, leading to elevated collision risks during operation. These constraints hinder their widespread deployment in complex and variable settings [30,31].

Advances in AI have facilitated the application of reinforcement learning (RL) in mobile robotics [32]. As a machine learning approach, RL demonstrates significant potential for use in autonomous robot decision-making [33]. It addresses key challenges including task execution policy optimization, environmental adaptability enhancement, and autonomous decision-making for complex tasks [34,35]. By learning optimal policies through environmental interaction, RL enables effective decision-making in dynamic and uncertain environments [36].

To enhance the time efficiency for mobile robot navigation in crowded environments, Zhou et al. [37] proposed a social graph-based double dueling deep Q-Network (DQN). This approach employs a social attention mechanism to extract effective graph representations, optimizes the state–action value approximator, and leverages simulated experiences generated by a learned environmental model for further refinement, demonstrating significantly improved success rates in crowded navigation tasks. Li et al. [38] introduced a fused deep deterministic policy gradient (DDPG) method, integrating a multi-branch deep learning network with a time-critical reward function, which effectively enhanced the convergence velocity and navigation performance in complex environments.

On the other hand, the control barrier function (CBF) has garnered attention in regard to obstacle avoidance control for mobile robots [39,40,41]. Originating from control theory, the CBF provides robust control guarantees for safety-critical systems operating under constraints [42]. For mobile robot obstacle avoidance, the CBF enforces strict safety conditions during motion by constructing safety constraints [43,44].

In order to further enhance the obstacle avoidance performance of the CBF, researchers have proposed various improvements and hybrid approaches. Singletary et al. [45] conducted a comparative analysis of the performance of the CBF and artificial potential fields (APFs) in robotic obstacle avoidance, demonstrating that the CBF generates smoother trajectories, effectively mitigates oscillations, and offers enhanced safety guarantees. Jian et al. [46] introduced a dynamic control barrier function, integrating it with model predictive control (MPC) to ensure collision-free trajectories for robots operating in uncertain and dynamic environments.

This paper proposes a novel safe reinforcement learning framework integrating the control barrier function (CBF) and proximal policy optimization (PPO) to reduce collisions in navigation for a mecanum wheel robot. Implemented within the ROS Gazebo simulation environment, the framework leverages CBF-based safety constraints formulated as a quadratic programming problem. This dynamically adjusts potentially unsafe actions generated by the PPO policy, guaranteeing collision mitigation while preserving motion efficiency. Comparative experiments against baseline algorithms (PPO, DQN, and DDPG) are conducted to rigorously evaluate the method’s effectiveness across key safety and navigation performance metrics.

The remainder of this paper is organized as follows: Section 2 introduces the robot model and basic methods; Section 3 presents the main research and methods; Section 4 describes the simulation results and and provides a discussion; and the final section, Section 5, concludes this paper.

## 2. Preliminary Knowledge

This section establishes the theoretical groundwork for the mobile robot platform, detailing its kinematic model and core implementation approaches. To support the development of subsequent control strategies and navigation algorithms, the unique omnidirectional motion capabilities inherent to the mecanum wheel robot are mathematically characterized. This section then presents fundamental obstacle avoidance techniques utilizing relevant control methodologies.

### 2.1. Kinematic Model of Robot

**Figure 1:**
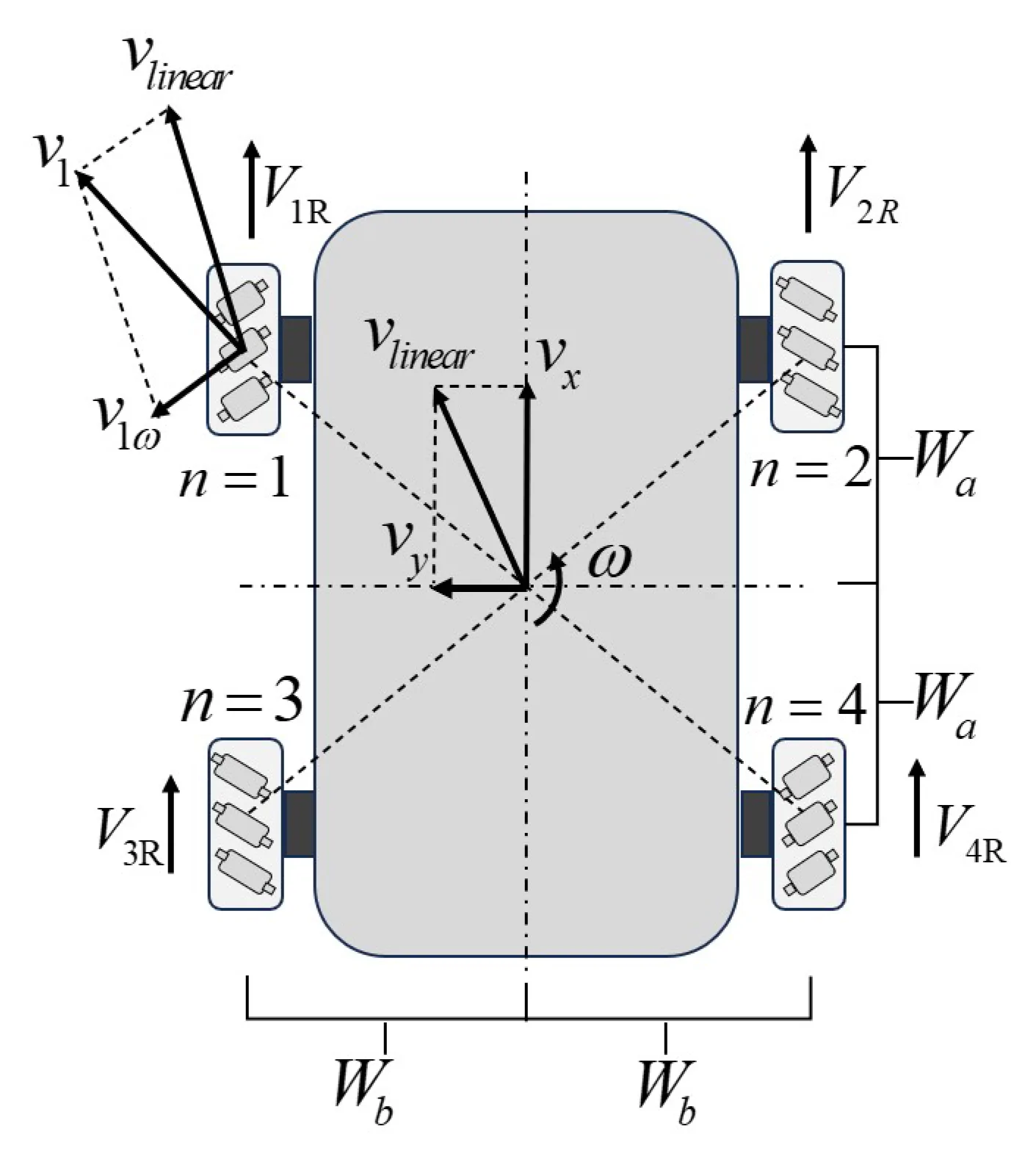
Kinematic model of mecanum wheel robot.

The mecanum wheel robot can achieve omnidirectional movement due to its unique wheel hub structure. The passive rollers installed at a 45° angle around each wheel allow the robot to translate or rotate in any direction without changing the orientation of the robot. Its kinematic model defines the relationship between the wheel velocities and the robot’s holistic motion. This representation is illustrated in Figure 1.

#### 2.1.1. Forward and Inverse Kinematics Models

The parameters related to the robot chassis are defined in Figure 1. In the model $W_{a}$ represents half of the body length (from the front wheel to the center of the rear wheel), and $W_{b}$ represents half of the body width (the distance between the left and right wheel centers). The robot’s linear velocity is $v_{linear}$, and $v_{x}$ and $v_{y}$ are the velocities in the *x* and *y* directions, respectively.

In addition $v_{n}\;\left(n=1,2,3,4\right)$ is the linear velocity of each wheel, and $v_{n\omega }$ is the angular velocity of the chassis corresponding to each wheel, and $V_{nR}$ represents the velocity at which the wheel moves forward. In summary, the forward kinematics model of the robot can be described as

(1) $$\left[\begin{array}{c} v_{x}\\ v_{y}\\ \omega \end{array}\right]=J{\left[\begin{array}{cccc} V_{1R} & V_{2R} & V_{3R} & V_{4R} \end{array}\right]}^{T},$$

(2) $$J=\frac{1}{4}\left[\begin{array}{cccc} 1 & 1 & 1 & 1\\ -1 & 1 & 1 & -1\\ -\frac{1}{d} & -\frac{1}{d} & \frac{1}{d} & \frac{1}{d} \end{array}\right],$$

where $d=W_{a}+W_{b}$ represents the characteristic length of the robot chassis. The matrix *J* establishes the mapping relationship between the wheel velocities and the chassis motion in the body-fixed frame. The corresponding inverse kinematics of the mecanum wheel robot chassis are shown as

(3) $$\left\{\begin{array}{c} V_{1R}=v_{x}-v_{y}-\left(W_{a}+W_{b}\right)\omega \\ V_{2R}=v_{x}+v_{y}+\left(W_{a}+W_{b}\right)\omega \\ V_{3R}=v_{x}+v_{y}-\left(W_{a}+W_{b}\right)\omega \\ V_{4R}=v_{x}-v_{y}+\left(W_{a}+W_{b}\right)\omega \end{array}\right..$$

**Figure 2:**
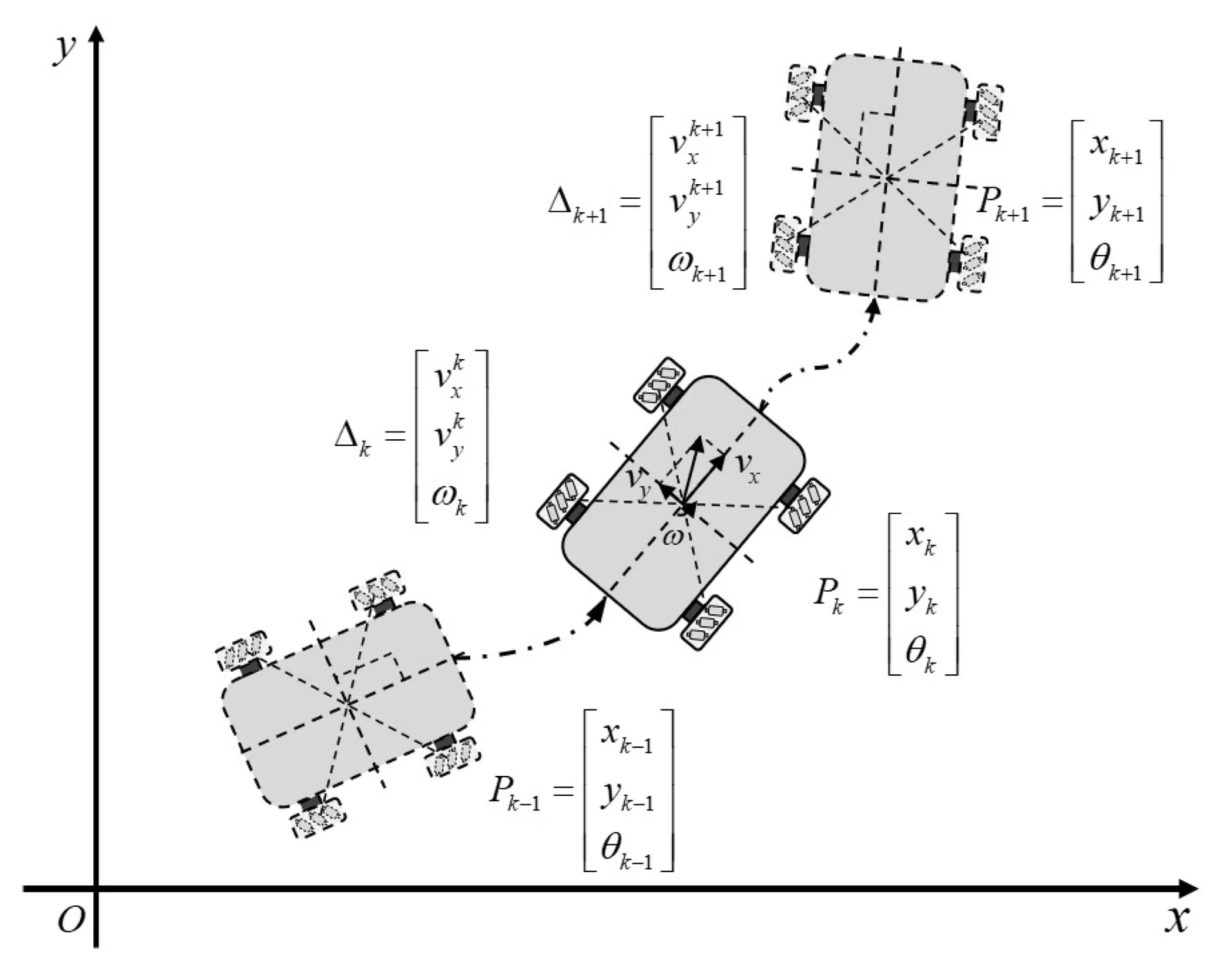
Wheel odometry model of robot.

Therefore, the velocity of the robot chassis and the velocity of the four wheels can be converted into each other using the forward and inverse kinematics, which facilitates control of the robot’s trajectory. Based on this, a schematic diagram of the robot’s wheel odometry model is shown in Figure 2.

#### 2.1.2. Ideal Model of Robot’s Odometry

Under ideal conditions, the relationship between the robot’s current position, $P_{k}$, and the previous position, $P_{k-1}$, is expressed by the offset $\Delta_{k}$. The next posture, $P_{k+1}$, is the current posture, $P_{k}$, plus the offset $\Delta_{k+1}$, and the corresponding expression equation is

(4) $$P_{k+1}=P_{k}+\Delta_{k+1}\Delta_{t+1},$$

where $P_{k}={\left[\begin{array}{ccc} x_{k} & y_{k} & \theta_{k} \end{array}\right]}^{T}$, and $\Delta_{k+1}={\left[\begin{array}{ccc} v_{x}^{k+1} & v_{y}^{k+1} & \omega_{k+1} \end{array}\right]}^{T}$. Assuming that the time, $\Delta_{t+1}$, between two points is very small, the next position can be expressed as the linear and angular velocity offsets of the current position, and the expanded component form is as follows:

(5) $$\left\{\begin{array}{cc} x_{k+1} & =x_{k}+v_{x}^{k+1}\\ y_{k+1} & =y_{k}+v_{y}^{k+1}\\ \theta_{k+1} & =\theta_{k}+\omega_{k+1} \end{array}\right..$$

Therefore, the derived forward and inverse kinematic models, combined with the discrete-time odometry update equations, provide a complete mathematical framework to predict the robot’s chassis motion based on the wheel velocities, which forms the basis for controlling the robot’s trajectory.

### 2.2. Control Barrier Function

In the process of robot movement and navigation, obstacle avoidance and velocity limiting are indispensable to prevent uncontrollable behavior. This section mainly introduces the control barrier function (CBF), which is often used in the control field as a controller and can also be used to constrain a robot’s actions.

#### 2.2.1. Definition of Safety Set

**Figure 3:**
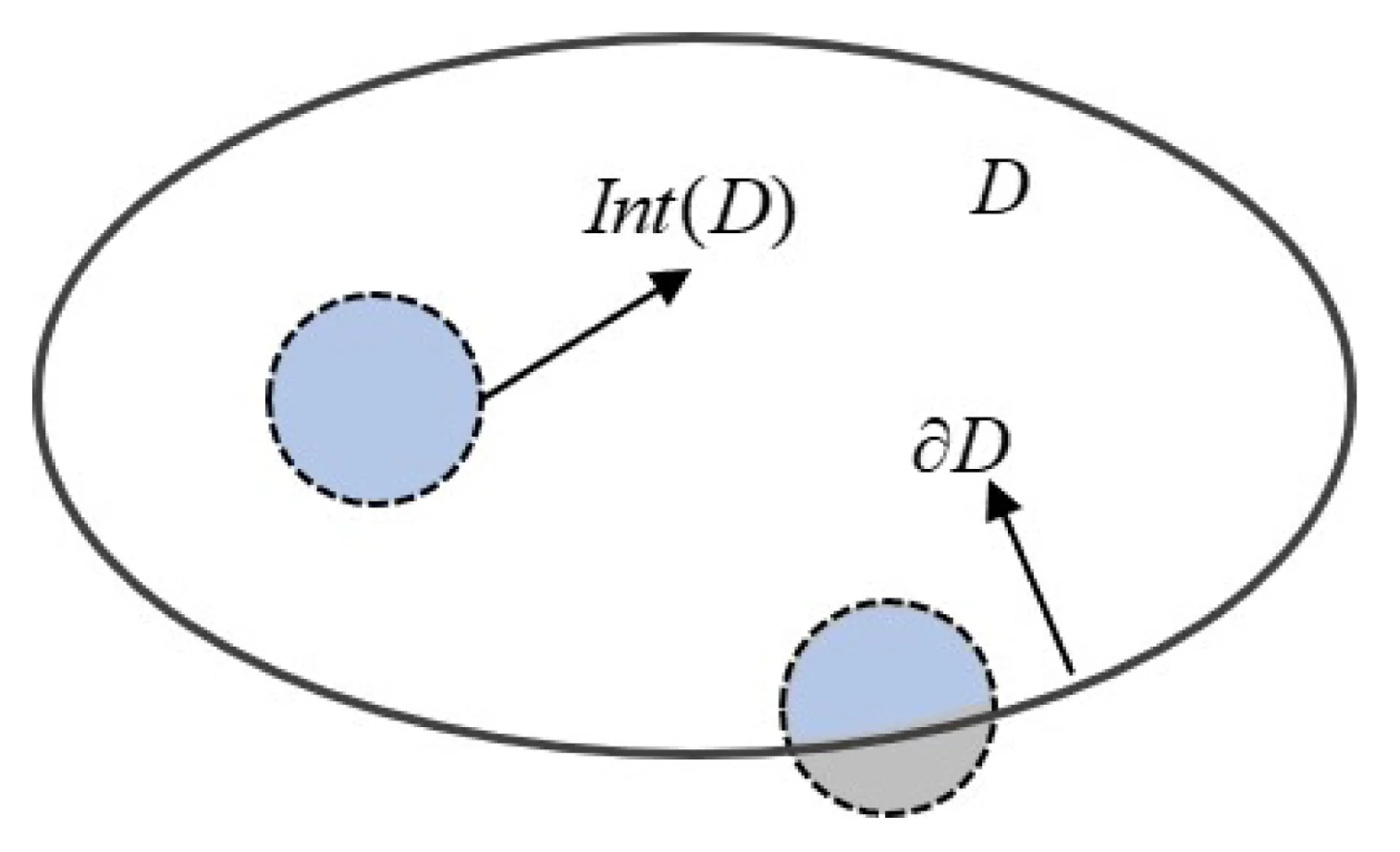
State space safety region visualization.

For a dynamic system, the safety set D is defined as the set of safe states in the system. A diagram of the safety set is shown in Figure 3, and D is defined as

(6) $$\begin{array}{cc} D & =\{x|x\in R^{n}\wedge h\left(x\right)\geq 0\},\\ \partial D & =\{x|x\in R^{n}\wedge h\left(x\right)=0\},\\ \mathrm{Int}(D) & =\{x|x\in R^{n}\wedge h\left(x\right)>0\}, \end{array}$$

where the safe set D contains the status $x\subset R^{n}$. When the system state *x* is safe, it means that it is inside the safety combination D or just on the boundary, which is h(x)≥0. On the contrary, h(x)<0 means that state *x* is outside the safe set.

#### 2.2.2. Definition of Control Barrier Funciton

Consider a control affine system and the corresponding dynamic system in the nonlinear case, which can be expressed as

(7) $$\dot{x}=F\left(x,u\right),$$

(8) $$\dot{x}=f\left(x\right)+g\left(x\right)u,$$

where $u\subset R^{n}$, and *F* is Lipschitz continuous. The behavior of the system is *f* when there is no control input, and *g* represents the control of the system and affects the changes in the system through a control input, *u*.

For h(x), ∀x∈D, and ∇h(x)≠0, there exists α, such that any state, *x*, in the set D satisfies the condition

(9) $$\dot{h}\left(x,u\right)=L_{f}h\left(x\right)+L_{g}h\left(x\right)u\geq -\alpha h\left(x\right),$$

where h(x) is considered to be a CBF, and α is an extended *K* function.

### 2.3. Basic Theory of Reinforcement Learning

Reinforcement learning (RL) is a machine learning approach characterized by an agent learning to optimize its actions through trial-and-error interactions with a dynamic environment, aimed at discovering strategies that maximize the long-term returns. Its core feature is learning through a trial-and-error mechanism, relying on reward signals to guide behavior optimization.

RL usually models a problem as a Markov decision process (MDP). A MDP can be represented by a five-tuple, S,A,P,R,γ.

*S*: The state space represents the set of all the possible states of the agent in the environment. The state x∈S represents a specific situation in the environment at a certain moment and contains all the information needed by the agent.

*A*: The Action Space and the actions that the agent can take during an interaction with the environment are usually represented by A(s), where a∈A(s) represents the set of possible actions in state *s*.

*P*: The State Transition Function represents the probability of transitioning to state $s_{t+1}$ if an action, *a*, is executed in state *s*: that is, $P(s_{t+1}|s_{t},a)$.

*R*: The reward function can be written as R(s,a) and refers to the immediate reward feedback obtained by the agent when it performs action *a* in state *s*.

γ: The discount factor is used to calculate future cumulative rewards, γ∈[0,1]. A larger discount factor is suitable for tasks that focus on achieving long-term goals, while a smaller discount factor is more suitable for tasks that emphasize immediate feedback.

**Figure 4:**
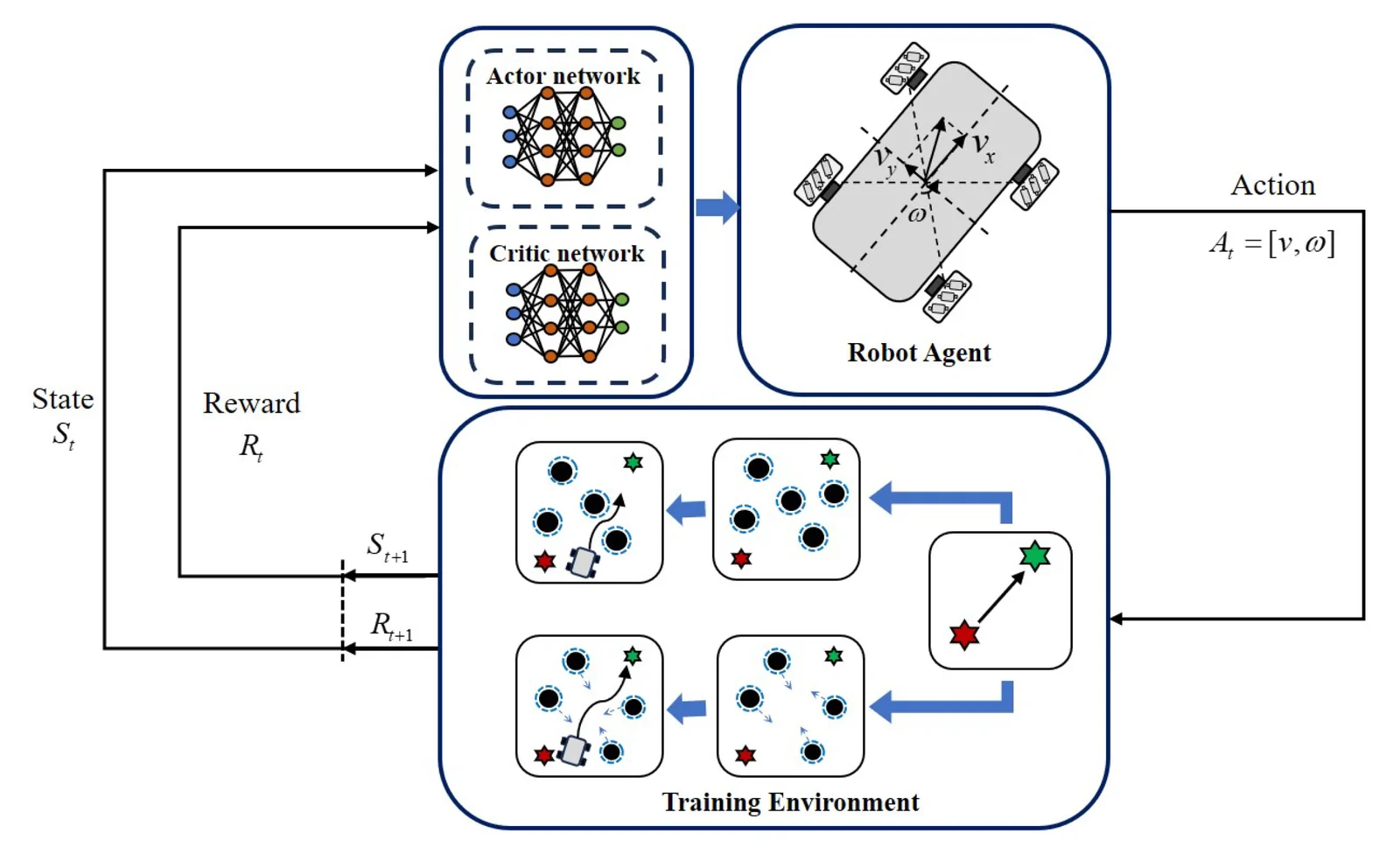
Training robot agent to interact with environment.

A robot can be regarded as an intelligent agent in reinforcement learning. The process of training it to interact with the environment involves RL. The corresponding environmental interaction is shown in Figure 4.

Figure 4 depicts the iterative learning process through which an agent learns to interact with its environment within an Actor–Critic framework. The Actor generates an action mechanism and communicates it to the robot agent. The robot explores the environment and obtains the corresponding rewards and states, which are then passed to the Critic network for value calculation. The Critic updates the Actor with the calculated parameters and outputs a better action strategy.

## 3. Main Research Content and Methods

This section mainly describes the motion control method based on proximal policy optimization (PPO) and the design of the real-time constraint mechanism using the CBF. On this basis, a novel fusion framework is constructed to implement a strategy for safe reinforcement learning, so as to ensure that the robot maintains safety performance while exerting its exploration advantages and further improve the stability of reinforcement learning.

### 3.1. Proximal Policy Optimization

The PPO objective function and Actor–Critic architecture are defined as key mechanisms for generating efficient exploratory navigation strategies for omnidirectional mobile robotic platforms. Their core function is to learn adaptive obstacle avoidance strategies for use in complex or dynamic scenarios and output robot velocity commands that balance navigation task completion and safety.

#### 3.1.1. PPO Principle Description

As an Actor–Critic derivative, PPO enforces policy update constraints via a trust-region-inspired mechanism to ensure monotonic improvement. By introducing an objective function clipping mechanism, it achieves efficient learning while ensuring training stability. Its core components and formulas are as follows:

(10) $$\mathrm{Actor}:\pi_{\theta }\left(a_{t}|s_{t}\right),$$

(11) $$\mathrm{Critic}:V_{\phi }\left(s_{t}\right),$$

where θ and ϕ are the parameters of the policy network and the value network, respectively, the current state is $s_{t}$, and $a_{t}$ is the generated action.

First, we need to calculate the generalized advantage estimation (GAE), calculate the TD error based on $V_{\phi }\left(s_{t}\right)$, and calculate the advantage $\hat{A}t$ based on the TD error $\delta_{t}$. The specific equation is expressed as

(12) $$\delta_{t}=r_{t}+\gamma V_{\phi }\left(s_{t+1}\right)-V_{\phi }\left(s_{t}\right),$$

(13) $$\hat{A}t=\delta_{t}+\gamma \lambda \delta_{t+1}+\ldots +{\left(\gamma \lambda \right)}^{T-t+1}\delta_{T-1},$$

with γ∈[0,1] being the discount factor and λ∈[0,1] the GAE parameter.

The importance sampling ratio in the policy network update $r_{t}\left(\theta \right)$ is

(14) $$r_{t}\left(\theta \right)=\frac{\pi_{\theta }\left(a_{t}|s_{t}\right)}{\pi_{\theta_{\mathrm{old}}}\left(a_{t}|s_{t}\right)}.$$

Next, we need to calculate the clipping objective function, which is also one of the core components of PPO. It can be expressed as

(15) $$L_{clip}^{PPO}\left(\theta \right)=E_{t}\left[\min\left(r_{t}\left(\theta \right){\hat{A}}_{t},\mathrm{clip}\left(r_{t}\left(\theta \right),1-\epsilon ,1+\epsilon \right){\hat{A}}_{t}\right)\right],$$

where ϵ is the clipping threshold, and it limits the importance sampling ratio $r_{t}\left(\theta \right)$ to within the interval [1−ϵ,1+ϵ].

For an action with a positive advantage $\left({\hat{A}}_{t}>0\right)$, the objective function is clipped to prevent the policy from increasing the probability of the action too aggressively $\left(r_{t}\left(\theta \right)>\left(1+\epsilon \right)\right)$. Conversely, for an action with a negative advantage $\left({\hat{A}}_{t}<0\right)$, the objective function is clipped to prevent the probability from decreasing too much $\left(r_{t}\left(\theta \right)<\left(1-\epsilon \right)\right)$.

In the Critic network update, the value loss function that needs to be calculated is as follows:

(16) $$L_{vf}^{PPO}\left(\phi \right)=Et\left[{\left(V\phi \left(s_{t}\right)-R_{t}\right)}^{2}\right],$$

where $R_{t}=\sum_{k=0}^{T}\gamma^{k}r_{t+k}$ is the cumulative return. At the same time, PPO introduces an entropy regularization term to enhance the exploration of strategies:

(17) $$L_{ent}^{PPO}\left(\theta \right)=Et\left[H(\theta \left(a|s_{t}\right))\right],$$

where $H(\theta \left(a|s_{t}\right))$ is the entropy of the strategy.

Then the total loss function can be calculated as

(18) $$L_{total}^{PPO}\left(\theta ,\phi \right)=E_{t}\left[L_{clip}^{PPO}\left(\theta \right)-c_{1}L_{vf}^{PPO}\left(\phi \right)+c_{2}L_{ent}^{PPO}\left(\theta \right)\right],$$

where $c_{1}$ and $c_{2}$ are weight parameters.

#### 3.1.2. PPO Algorithm Flow

The PPO algorithm is an efficient policy gradient method. By introducing an objective function clipping mechanism, it can achieve efficient learning while ensuring training stability. The core idea of PPO is to limit the step size of policy updates to avoid performance crashes during training. The algorithm flow is shown in Algorithm 1.
**Algorithm 1** Proximal policy optimization strategy.**Input:** Initial policy $\theta_{0}$, value $\phi_{0}$, ϵ, γ, λ, $c_{1}$, $c_{2}$, *K*, *M***Output:** Optimized $\theta^{*}$, $\phi^{*}$
1:Initialize $\theta \leftarrow \theta_{0}$, $\phi \leftarrow \phi_{0}$2:**for** k=0 **to** *K* **do**3:    Collect $D_{k}$ via $\pi_{\theta }$4:    **for** each *t* in $D_{k}$ **do**5:        $R_{t}\leftarrow \sum_{l=t}^{T}\gamma^{l-t}r_{l}$6:        ${\hat{A}}_{t}\leftarrow \sum_{l=0}^{T-t}{\left(\gamma \lambda \right)}^{l}\delta_{t+l}$7:    **end for**8:    $\theta_{old}\leftarrow \theta$9:    **for** episode =1 **to** *M* **do**10:        Sample minibatch B from $D_{k}$11:        $L_{total}^{PPO}\left(\theta ,\phi \right)\leftarrow 0$12:        **for** each $\left(s_{t},a_{t},R_{t},{\hat{A}}_{t}\right)$ in B **do**13:           $r_{t}\left(\theta \right)\leftarrow \pi_{\theta }\left(a_{t}|s_{t}\right)/\pi_{\theta_{old}}\left(a_{t}|s_{t}\right)$14:           $L_{clip}^{PPO}\left(\theta \right)\leftarrow \min\left(r_{t}\left(\theta \right){\hat{A}}_{t},\mathrm{clip}\left(r_{t}\left(\theta \right),1-\epsilon ,1+\epsilon \right){\hat{A}}_{t}\right)$15:           $L_{vf}^{PPO}\left(\phi \right)\leftarrow {\left(V_{\phi }\left(s_{t}\right)-R_{t}\right)}^{2}$16:           $L_{ent}^{PPO}\left(\theta \right)\leftarrow -Et\left[H(\theta \left(a|s_{t}\right))\right]$17:           $L_{total}^{PPO}\left(\theta ,\phi \right)\leftarrow L_{total}^{PPO}\left(\theta ,\phi \right)-L_{clip}^{PPO}\left(\theta \right)+c_{1}L_{vf}^{PPO}\left(\phi \right)-c_{2}L_{ent}^{PPO}\left(\theta \right)$18:        **end for**19:        $\theta^{*}\leftarrow \theta -\eta_{\theta }\nabla_{\theta }L_{total}^{PPO}$20:        $\phi^{*}\leftarrow \phi -\eta_{\phi }\nabla_{\phi }L_{total}^{PPO}$21:    **end for**22:**end for**23:**return** $\theta^{*}\leftarrow \theta$, $\phi^{*}\leftarrow \phi$

### 3.2. Application and Implementation of CBF

The CBF is specifically used for its safety conditions, which are based on the kinematic model of the omnidirectional mobile robot used in this study. The focus is on demonstrating how this function acts as a real-time safety filter, applying online corrections to the original motion output by the PPO algorithm to achieve collision avoidance during navigation. Its quadratic programming formulation explicitly serves a single goal: to minimize the safety corrections to the PPO-determined motion while ensuring that the robot maintains its specific safety distance and stays within its physical motion limits.

#### 3.2.1. Safe Sets and Safe Functions

The set of states, D, in which the robot can safely operate and the barrier function h(p) are defined as

(19) $$D=\{p\subset R^{n}:h\left(p\right)\geq 0\},$$

(20) $$h\left(p\right)=||p-p_{obs}{\left|\right|}^{2}-r_{safe}^{2},$$

where p=[x,y] represents the coordinates of the robot in the global coordinate system, $p_{obs}$ represents the coordinates of the nearest obstacle, and $r_{safe}$ is the safety distance threshold.

#### 3.2.2. Implementation of CBF Safety Constraints

The time derivative of the CBF can be calculated as

(21) $$\begin{array}{cc} \frac{dh}{dt} & =\frac{\nabla h\cdot dp}{dt}\\ & =\left[\frac{\partial h}{\partial x},\frac{\partial h}{\partial y}\right]\cdot {\left[\dot{x},\dot{y}\right]}^{T}, \end{array}$$

where $\frac{\partial h}{\partial x}=2\left(x-x_{obs}\right)$, $\frac{\partial h}{\partial y}=2\left(y-y_{obs}\right)$. Then substitute the robot motion model into the equation

(22) $$\frac{dh}{dt}=2v\left[\left(x-x_{obs}\right)cos\theta +\left(y-y_{obs}\right)sin\theta \right].$$

Since the CBF needs to satisfy this constraint, substitute Equation (22) into Equation (9):

(23) $$2v\left[\left(x-x_{obs}\right)cos\theta +\left(y-y_{obs}\right)sin\theta \right]\geq -\alpha \left({\left(x-x_{obs}\right)}^{2}+{\left(y-y_{obs}\right)}^{2}-r_{safe}^{2}\right).$$

Set a relative position vector, Δp, and the unit vector of the robot’s forward direction, *i*, as

(24) $$\Delta p={\left[\begin{array}{cc} x-x_{obs}, & y-y_{obs} \end{array}\right]}^{T},$$

(25) $$i={\left[\begin{array}{cc} \cos\theta , & \sin\theta \end{array}\right]}^{T}.$$

The corresponding constraints can be transformed into

(26) $$2v\cdot \left(\Delta p\cdot i\right)\geq -\alpha (∥\Delta p{∥}^{2}-r_{\mathrm{safe}}^{2}).$$

In summary, the overall constraint solving framework of the CBF can be written as

(27) $$\begin{array}{cc} {\left(v,\omega \right)}^{*}=\arg\min_{v,\omega \in R^{n}} & \left[{\left(v-a_{t}\left(v\right)\right)}^{2}+{\left(\omega -a_{t}\left(\omega \right)\right)}^{2}\right]\\ s.t. & \;2v\cdot \left(\Delta p\cdot n\right)\geq -\alpha (∥\Delta p{∥}^{2}-r_{safe}^{2}),\\ \; & \;0\leq v\leq v_{\max},\\ \; & \;|\omega |\leq \omega_{\max}, \end{array}$$

where $v_{max}$ is the max value of *v*, and $\omega_{max}$ is the max angular velocity of ω.

**Figure 5:**
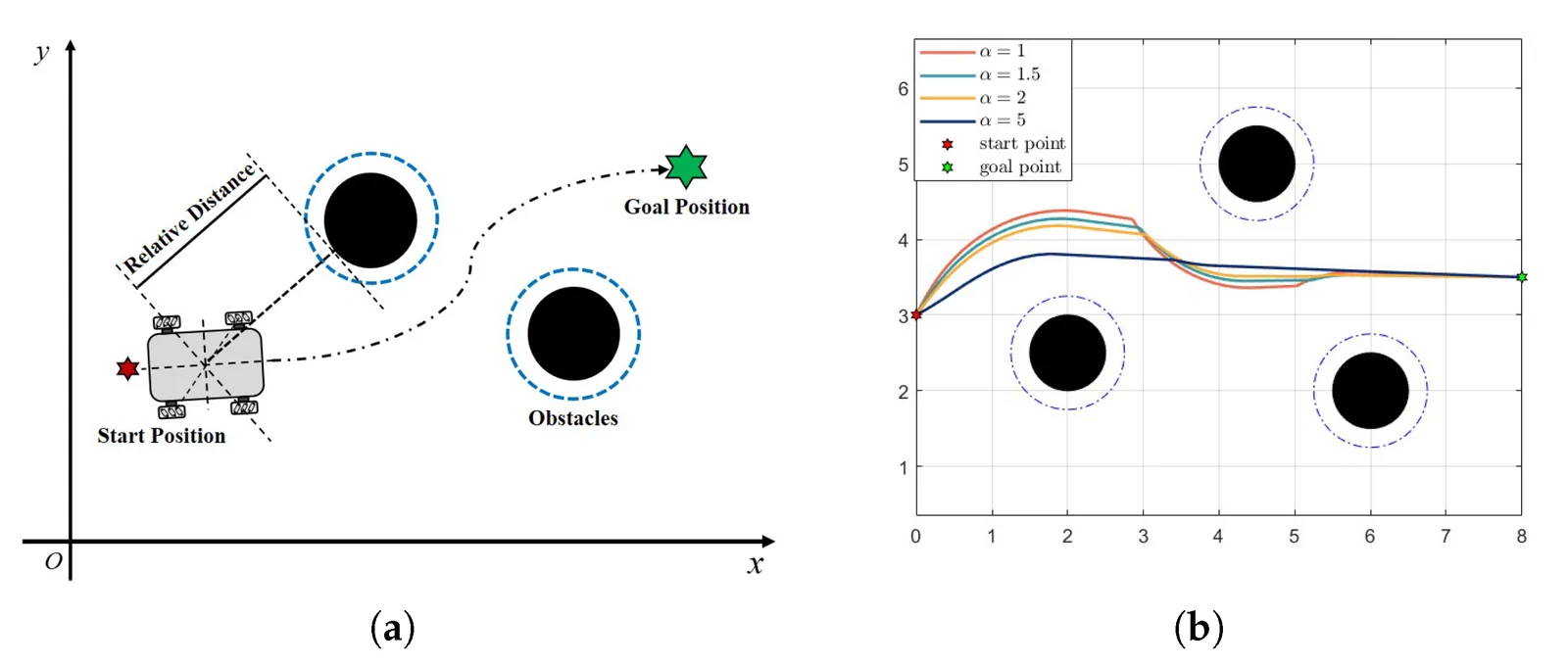
CBF example: (**a**) Sequential points showing the robot’s navigation path through an environment containing obstacles. (**b**) Comparative analysis of the trajectory variations under different α parameter values.

Figure 5 shows a simple diagram of the CBF obstacle avoidance process, as well as an example of the impact of different α values on the trajectory. At each step, the CBF combines the current velocity and other state variables to determine the next output that meets the constraint requirements and combines this with the result from the obstacle function calculation to continuously move until it reaches the target position.

A CBF framework for safe robot motion is proposed. First, a safe set is used by a barrier function, which represents the set of collision-free trajectories. Then, the time derivative of this barrier function is derived from the robot’s kinematic model and combined with the *K* function to obtain linear inequalities for the control variables. Finally, a quadratic program is formulated to minimize the modification of the nominal control command while satisfying the CBF safety constraints and the actuator’s restrictions on the linear and angular velocities. The corresponding algorithm for the CBF is shown in Algorithm 2.
**Algorithm 2** CBF safety filter.**Input:** Robot kinematic model, safety distance threshold $r_{safe}$, maximum velocity $v_{\max}$, maximum angular velocity $\omega_{\max}$, CBF parameters α**Output:** Optimized control inputs $v^{*}$, $\omega^{*}$
1:Initialize robot state p=[x,y], robot orientation θ, velocity *v*, and angular velocity ω2:Calculate the relative position vector $\Delta p={\left[x-x_{obs},y-y_{obs}\right]}^{T}$ to the nearest obstacle3:Calculate the unit vector of the robot forward direction $i={\left[\cos\theta ,\sin\theta \right]}^{T}$4:Calculate the barrier function $h\left(p\right)={∥\Delta p∥}^{2}-r_{safe}^{2}$5:Calculate the time derivative of the barrier function using (22)6:Set the initial control inputs $v_{0}$, $\omega_{0}$7:**for** k=0 **to** *K* **do**8:       Calculate the constraint $2v\cdot \left(\Delta p\cdot i\right)\geq -\alpha (∥\Delta p{∥}^{2}-r_{safe}^{2})$9:      where $i={\left[\cos\theta ,\sin\theta \right]}^{T}$10:    and $\Delta p={\left[x-x_{obs},y-y_{obs}\right]}^{T}$11:  Solve the optimization problem$$\begin{array}{cc} {\left(v,\omega \right)}^{*}=\arg\min_{v,\omega \in R^{n}} & \left[{\left(v-a_{t}\left(v\right)\right)}^{2}+{\left(\omega -a_{t}\left(\omega \right)\right)}^{2}\right]\\ s.t. & \;2v\cdot \left(\Delta p\cdot i\right)\geq -\alpha (∥\Delta p{∥}^{2}-r_{safe}^{2}),\\ \; & \;0\leq v\leq v_{\max},\\ \; & \;|\omega |\leq \omega_{\max}. \end{array}$$
12:    Update control inputs $v^{*}\leftarrow v$, $\omega^{*}\leftarrow \omega$13:    Update robot state using the kinematic model14:**end for**15:**return** $v^{*}\leftarrow v$, $\omega^{*}\leftarrow \omega$

### 3.3. Risk-Aware, Dynamic, Adaptive Regulation Barrier Policy Optimization

**Figure 6:**
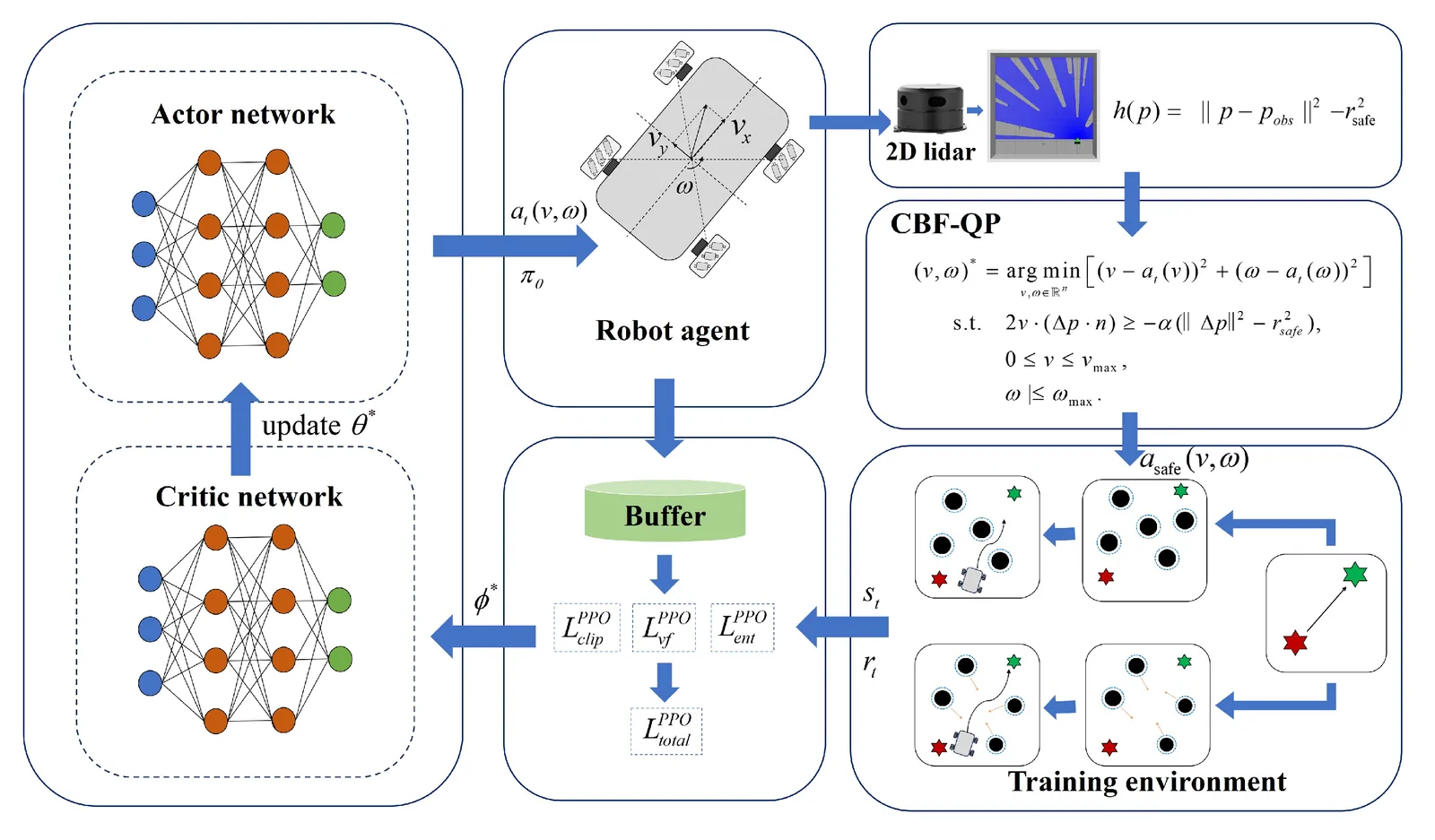
Complete RADAR-BPO framework for training to interact with environment.

This section designs a risk-aware, dynamic, adaptive regulation barrier policy optimization (RADAR-BPO) method which combines the efficiency of stable exploration with PPO. The Actor outputs probabilistic actions, and the Critic updates their value and optimizes the Actor based on the information obtained from feedback on the agent’s motion in the environment. In addition, extra security is provided by the CBF, which was added to further optimize the actions output by the PPO algorithm for the actor, filter actions with risky values, and output safe actions. This method deeply integrates the exploration capabilities of PPO and the security of the CBF, so that the robot agent can maximize the value of exploration in different environments. A corresponding block diagram of the complete system is shown in Figure 6, and the algorithm is shown in Algorithm 3.

**Algorithm 3** RADAR-BPO.**Input:** Initial policy $\theta_{0}$, value $\phi_{0}$, safety params**Output:** Optimized $\theta^{*}$, $\phi^{*}$
1:Initialize $\theta \leftarrow \theta_{0}$, $\phi \leftarrow \phi_{0}$2:**for** iteration k=0 **to** *K* **do**3:    **Data Collection:**4:    Collect trajectory $D_{k}$ via policy $\pi_{\theta }$5:    **Policy Optimization:**6:    Compute advantages ${\hat{A}}_{t}$ and returns $R_{t}$ for $D_{k}$7:    $\theta_{\mathrm{old}}\leftarrow \theta$8:    Update θ,ϕ using PPO loss with $D_{k}$9:    **for** each state $s_{t}$ in $D_{k}$ **do**10:        Get PPO action $a_{t}^{\mathrm{PPO}}=\left(v^{\mathrm{PPO}},\omega^{\mathrm{PPO}}\right)$11:        Compute safety constraint based on robot state12:        **Safety Filtering:**13:        Solve CBF-QP:$$\begin{array}{cc} \left(v^{*},\omega^{*}\right) & =\arg\min_{v,\omega }{∥a-a_{t}^{\mathrm{PPO}}∥}^{2}\\ s.t. & \;\;\mathrm{CBF}\;\mathrm{constraints} \end{array}$$14:        Execute safe action $a_{t}^{\mathrm{safe}}=\left(v^{*},\omega^{*}\right)$15:    **end for**16:**end for**17:**return** $\theta^{*}\leftarrow \theta$, $\phi^{*}\leftarrow \phi$

In addition, this method fully considers information on the robot’s spatial dimensions during the obstacle avoidance process, including its position, orientation, distance from obstacles, etc., and transforms this spatial information into safety constraints using the CBF and embeds it into a reinforcement learning-based decision-making process, thereby achieving safe and efficient navigation in complex dynamic environments.

To achieve an optimal balance between navigation efficiency and safety guarantees, a multi-objective reward function with adaptive components is designed. The mathematical formulation is defined as follows:

(28) $$r_{t}=r_{h}+r_{d}+r_{\mathrm{ob}}+r_{v},$$

where $r_{h}$, $r_{d}$, $r_{\mathrm{ob}}$, and $r_{v}$ are defined below.

The heading reward $r_{h}$ encourages alignment with the target direction:

(29) $$r_{h}=1+\cos\left(\theta_{diff}\right),$$

where $\theta_{diff}$ denotes the angular deviation between the robot’s current orientation and the target direction. The distance reward $r_{d}$ motivates progression toward the target position:

(30) $$r_{d}=5\exp\left(-2\frac{d_{t}}{d_{0}}\right),$$

where $d_{t}$ is the current Euclidean distance to the target and $d_{0}$ is the initial distance.

The obstacle penalty $r_{\mathrm{ob}}$ ensures collision risk awareness:

(31) $$r_{\mathrm{ob}}=\left\{\begin{array}{cc} r_{risk} & \mathrm{if}\;d_{\min}<r_{safe}\\ r_{normal} & \mathrm{otherwise}, \end{array}\right.$$

where $d_{\min}$ is the distance to the nearest obstacle.

The velocity reward $r_{v}$ optimizes the motion efficiency:

(32) $$r_{v}=\exp\left(-\frac{{\left(v_{t}-v_{des}\right)}^{2}}{2\sigma^{2}}\right),$$

with an adaptive optimal velocity of $v_{des}=0.25\min\left(1.0,d_{t}\right)$ and σ=0.1. This equation encourages a higher velocity when distant from the target while promoting precision during the final phases.

The reward function integrates the four components in an additive manner. Although this is an equally weighted sum, each term is designed to have comparable magnitudes to prevent any one objective from overly dominating the learning process. For example, $r_{h}\in \left[0,2\right]$, and $r_{d}$ is scaled by a factor and exponentially decays with distance. $r_{\mathrm{ob}}$ provides a significant but bounded penalty. This design prioritizes simplicity, interpretability, and minimal parameter tuning in the early stages of the algorithm’s implementation, resulting in a reliable benchmark. The $r_{v}$ component is not a constant reward but a term used to fine-tune the efficiency of the movement.

The RADAR-BPO framework establishes a safety-critical reinforcement learning paradigm through integrated policy optimization and real-time safety assurance. During each training iteration, the agent executes interactions with the environment using its current policy network $\pi_{\theta }$, gathering trajectory data that includes environmental states, exploratory actions, and reward signals. This collected experience forms the foundation for subsequent policy refinement.

Following data collection, the algorithm performs proximal policy optimization using the GAE. This involves calculating the temporal difference errors to estimate the action advantages, then updating both the policy and value networks using a specialized objective function. The optimization balances exploration incentives with value approximation while maintaining training stability through gradient clipping.

During action execution, each policy-generated velocity command undergoes safety verification using a CBF filter. This module solves a quadratic optimization problem that minimizes deviations from the original actions while enforcing collision avoidance constraints. The safety verification incorporates the robot’s positional relationship with obstacles and its current heading orientation to dynamically adjust the motion commands.

The resulting safe actions preserve the learning direction of the policy network while ensuring formal safety guarantees through real-time constraint enforcement. This synergistic integration creates an evolution mechanism where policy improvement and safety assurance mutually reinforce each other throughout the learning process. The system maintains continuous compliance with safety boundaries while progressively refining its navigation strategy through environmental interactions.

## 4. Case Study

To rigorously evaluate the performance and validate the efficacy of the proposed risk-aware, dynamic, adaptive regulation barrier policy optimization (RADAR-BPO) framework for collision risk mitigation in mobile robot navigation, this section presents comprehensive simulation experiments conducted within the ROS Gazebo environment.

### 4.1. Test Environment Setup

**Figure 7:**
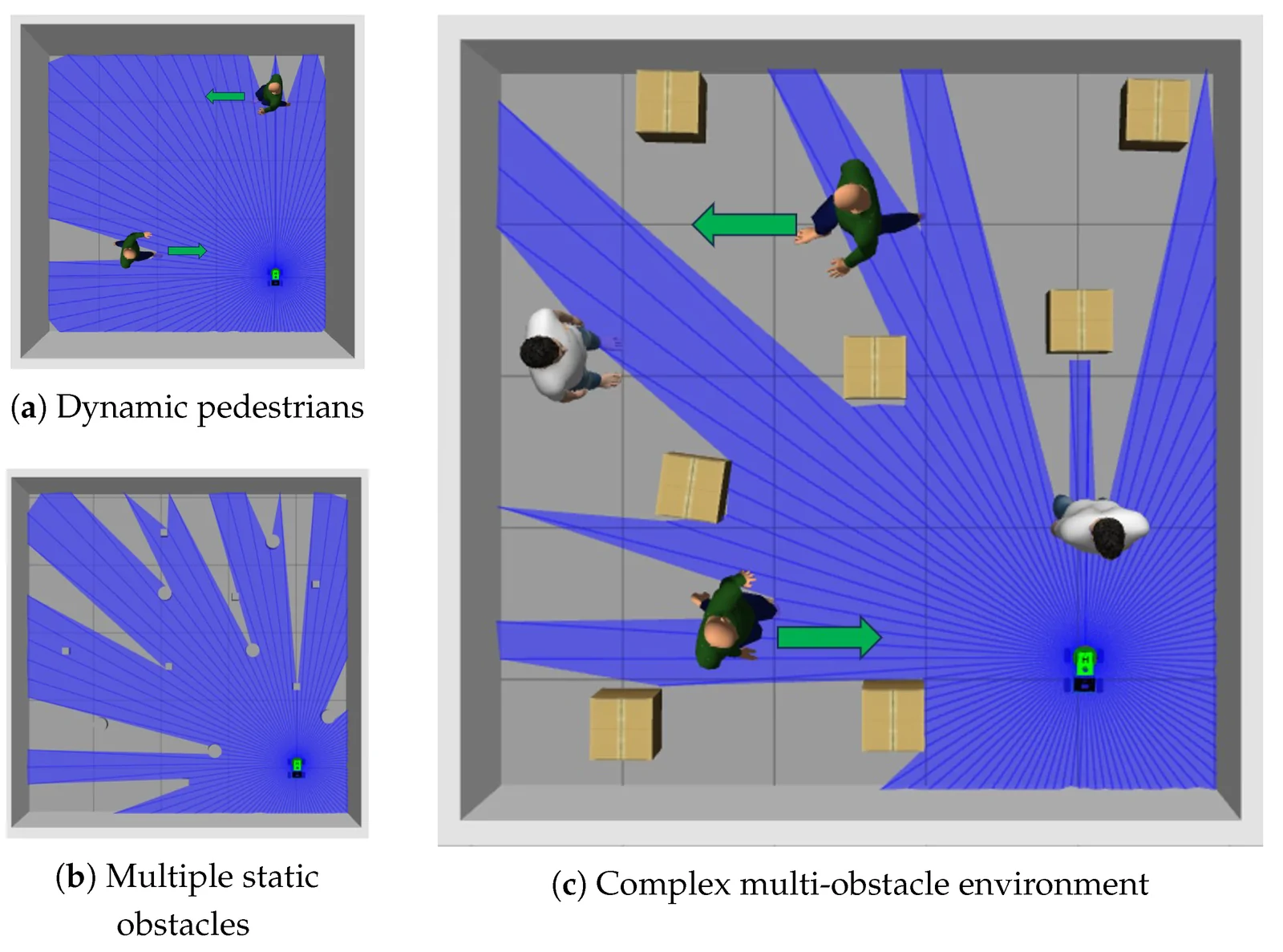
Simulation in three different environments.

The simulation environments visualized in Figure 7 were constructed within the ROS Gazebo platform to test a robot’s obstacle avoidance navigation. Specifically, Figure 7a presents a dynamic pedestrian environment, Figure 7b demonstrates a multi-obstacle environment, and Figure 7c showcases a complex obstacle environment incorporating both static and dynamic elements.

In Figure 7a, there are two pedestrians walking back and forth at a certain speed. The robot needs to avoid them and reach the target point while the pedestrians are moving. In Figure 7b, there are multiple static cylindrical or cubic obstacles. The robot needs to avoid the static obstacles and successfully navigate to the target point. Figure 7c contains multiple pedestrians and multiple different static obstacles. The robot also needs to complete a navigation task in this complex and changing environment.

### 4.2. Design of Experimental Test

The motion trajectories of the pedestrians in Figure 7a,c are shown in Table 1, and there are two stationary pedestrians in Figure 7c.

In the Gazebo coordinate system (the positive direction on the y-axis is left and the negative direction is right), pedestrian 1 moves 1 m to the right from their initial position (0.5, 2.5) to (0.5, 1.5), turns on the spot, moves 1 m to the left, returns to their starting point (0.5, 2.5), and stays there. At the same time, pedestrian 2 moves 2 m to the left from their initial position (3.0, 0.0) to (3.0, 2.0), turns on the spot, moves 2 m to the right, returns to their starting point (3.0, 0.0), and stays there. Both pedestrians complete a round trip in the y-axis direction. There are two other stationary pedestrians: one is fixed at (1.0, 0.0), and the other is fixed at (2.0, 3.0). The robot starts from the starting point (0.0, 0.0) and needs to traverse the dynamic environment to reach the target point (3.0, 3.0). Its path will be blocked by moving pedestrians and it will need to avoid stationary pedestrian obstacles and other obstacles.

Table 2 lists the key parameter settings used to train the RADAR-BPO navigation algorithm. These parameters cover aspects such as policy optimization, the safety constraints, the robot’s kinematic model, and the training environment configuration. Some parameter values (such as discount factors and GAE parameters) were within typical ranges or needed to be adjusted according to the specific environment.

The algorithm was trained on each environment for a fixed number of episodes: 150 for Env 1 (dynamic pedestrians), 200 for Env 2 (multiple static obstacles), and 300 for Env 3 (multiple complex obstacles). This design with an increasing environment complexity and training time was intended to mimic curriculum learning, allowing the agent to consolidate foundational skills before tackling more difficult tasks. Each training episode terminated when the agent successfully reached the goal, collided with an obstacle, or reached the maximum step limit.

### 4.3. Test Results and Analysis

To comprehensively evaluate the performance of the proposed RADAR-BPO framework and quantitatively assess its effectiveness in mitigating collision risks while maintaining navigation efficiency, extensive simulations were conducted across the three distinct environments introduced in Section 4.1 (Figure 7). The evaluation employed a three-stage progressive training paradigm: Stage 1 (Figure 7a) utilized the dynamic pedestrian environment (Env 1), Stage 2 (Figure 7b) progressed to the multi-static obstacle environment (Env 2), and Stage 3 (Figure 7c) took place in the complex hybrid environment containing both static obstacles and dynamic pedestrians (Env 3). This staged approach rigorously tested the algorithm to determine its fundamental obstacle avoidance ability.

**Figure 8:**
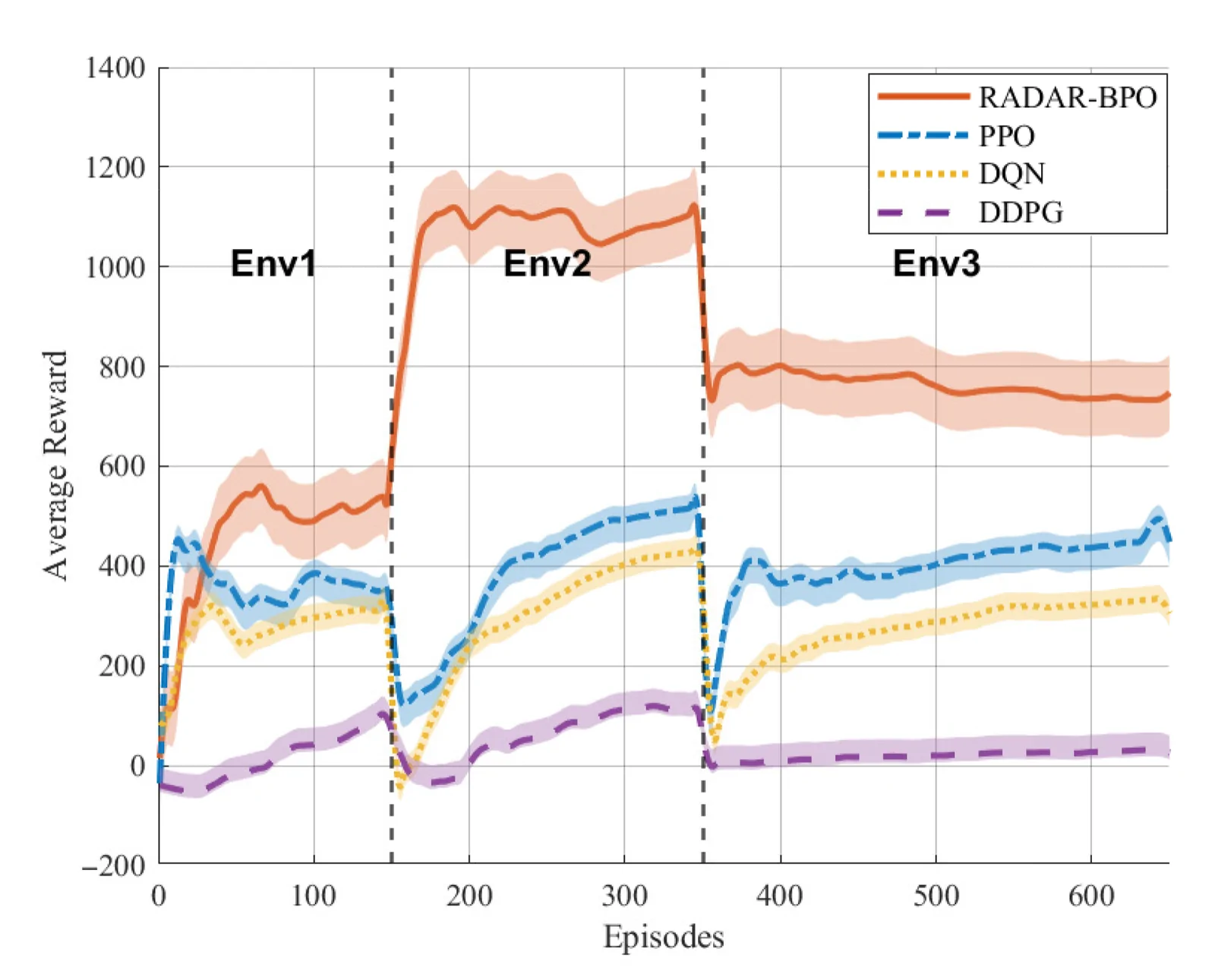
Reward curve resulting from three-stage training process.

The core performance metrics included the learning stability, safety performance, and navigation efficiency. Crucially, the cumulative reward curves from throughout the training process were analyzed and compared against those of the baseline PPO algorithm. This direct comparison highlighted the impact of integrating the real-time CBF safety filter within the RADAR-BPO framework. The corresponding segmented training reward curve is shown in Figure 8.

As shown in Figure 8, during the progressive training process implemented across three stages (Env 1, Env 2, and Env 3), the RADAR-BPO algorithm significantly outperformed all the baseline algorithms (PPO, DQN, and DDPG) in terms of the average reward in all the environments. This overall performance advantage was demonstrated by the following: in the relatively simple Env 1 stage, while all the algorithms initially experienced low rewards, RADAR-BPO quickly escaped this low-reward zone and stabilized at a higher level. Entering the more complex Env 2 stage, RADAR-BPO exhibited a burst of performance growth, with its reward values rapidly exceeding 1000 and stabilizing at approximately 1100. In contrast, PPO slowly climbed to around 400, while the DQN and DDPG lagged significantly behind. In the most complex Env 3 stage, the challenging environment caused performance degradation for all the algorithms. However, RADAR-BPO maintained an absolute advantage, stabilizing at around 760 with a smooth, volatility-resistant curve. PPO fluctuated violently below 400 and showed weak growth. The DQN and DDPG’s performance remained sluggish, failing to improve significantly, and the gap between their performance and RADAR-BPO’s was the largest.

Comparing the two curves at the beginning of the Env 3 phase reveals that PPO’s reward values exhibited a larger upward gradient and oscillation amplitude, while the rise in RADAR-BPO’s values was more stable. This phenomenon reveals the different learning modes of the two algorithms: The PPO strategy almost failed after an environment switch, requiring a painstaking learning process to recover from an extremely high collision rate, resulting in an unstable learning process. In contrast, RADAR-BPO benefited from the real-time safety guarantees provided by the CBF. Its strategy retained its core obstacle avoidance capabilities after an environment switch, and its learning process involved stable fine-tuning based on a higher performance baseline to adapt to new dynamic obstacles. Therefore, RADAR-BPO sacrificed a seemingly larger learning amplitude in exchange for a higher, more stable, and safer final performance.

This result clearly shows that the RADAR-BPO framework integrated with real-time CBF security filtering can not only achieve higher task returns in complex dynamic environments (based on the higher success rate and average reward in Table 3) but also significantly improve the convergence speed of the learning process, the final performance ceiling, and the training stability, effectively solving the core problems of low exploration efficiency, large policy fluctuations, and limited performance of traditional PPO in safety-critical scenarios.

According to the results listed in Table 3, RADAR-BPO outperformed the PPO algorithm in most metrics. In Env 1, RADAR-BPO’s collision rate was 68.67%, lower than PPO’s 82.00%; in Env 3, RADAR-BPO’s collision rate further decreased to 10.67%, compared to PPO’s 30.67%. Furthermore, RADAR-BPO achieved 47, 164, and 268 successes in the three different environments, respectively, all exceeding PPO’s 27, 134, and 208. In terms of the average reward, RADAR-BPO also generally outperformed PPO, with these algorithms achieving 1070.09 and 377.01, respectively, in Env 2. These results demonstrate that RADAR-BPO maintains high task completion efficiency and a high reward yield while reducing the collision rate.

In contrast, the DDPG and DQN algorithms generally underperformed in comparison to RADAR-BPO and PPO. The DDPG’s collision rate was above 75% across all the environments, and its average reward was significantly lower than that of the other algorithms. The DQN had zero successes and a 100% collision rate in Env 1. In Env 2 and Env 3, its collision rates were 55.50% and 68.00%, respectively. While its average reward was higher than that of the DDPG, it was still lower than that of RADAR-BPO and PPO.

To provide an intuitive visualization of the algorithm’s real-time decision-making and safety assurance capabilities in the most challenging scenario, the trajectory evolution of the robot navigating across Env 3 over a critical 9 s interval (from *t* = 1 s to *t* = 9 s) is presented and discussed. This sequence illustrates how RADAR-BPO dynamically adjusted the robot’s path to safely avoid both static obstacles and moving pedestrians while progressing towards the target.

**Figure 9:**
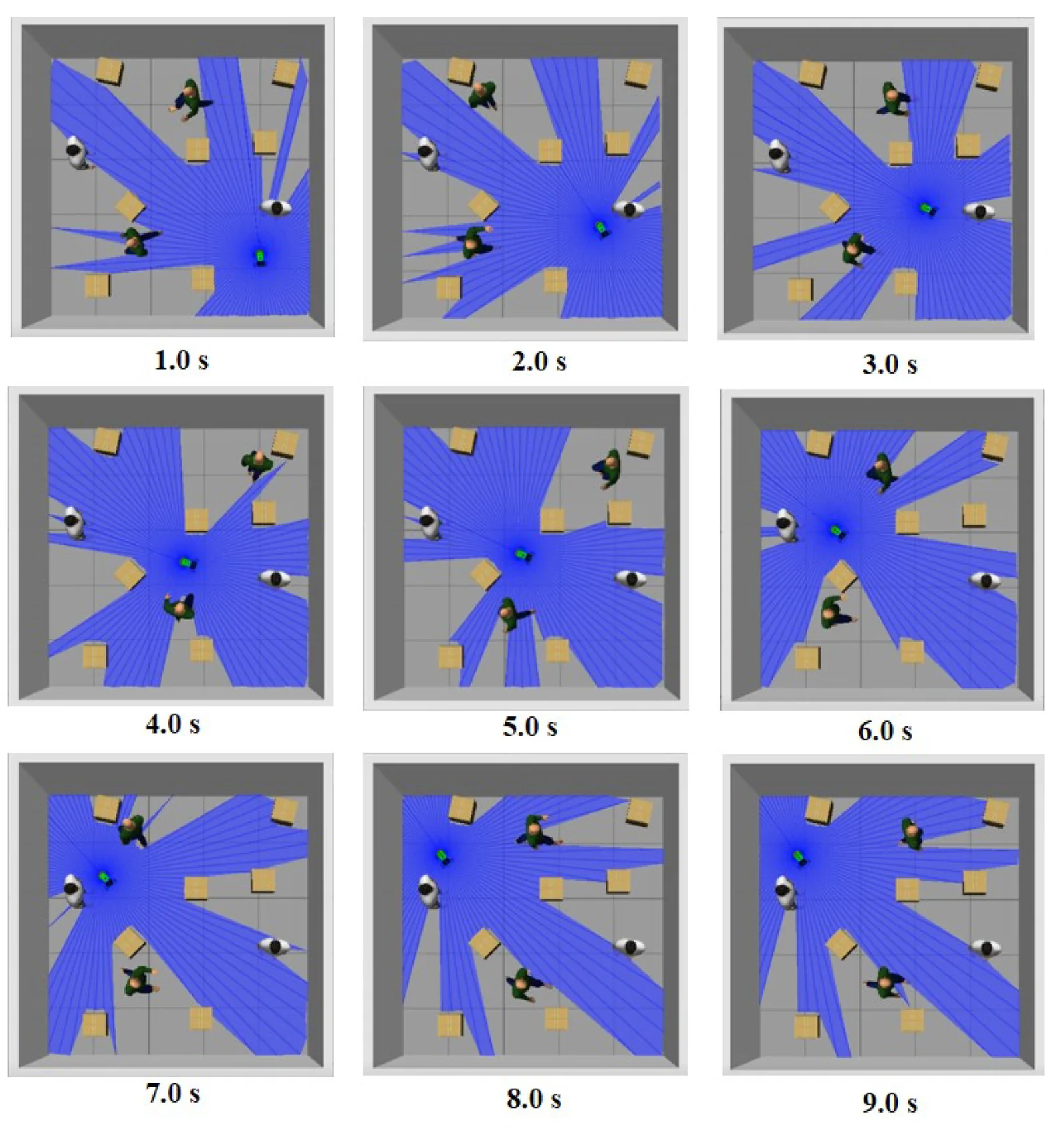
Trajectory of robot in complex hybrid environment.

In Figure 9, the robot starts from the starting position (0.0, 0.0), initially accelerates to bypass the stationary pedestrian in front, and turns left toward the target direction; when it encounters Pedestrian 1 (0.5, 2.5 → 1.5) moving horizontally to the right, it suddenly turns at a sharp angle to achieve emergency avoidance and simultaneously and smoothly avoids walls and nearby obstacles. Under the combined threat of Pedestrian 2 (3.0, 2.0 → 0.0) moving horizontally and dense obstacles, the robot flexibly adjusts the path to squeeze through and finally arrives at the end point (3.0, 3.0) accurately. The entire path maintains the straight-line efficiency of PPO in open areas and forms a smooth and conservative contour using CBF constraints when approaching obstacles. The collision-free nature of the entire process verifies RADAR-BPO’s seamless integration of exploration and safety in dynamic and dense scenes.

## 5. Conclusions and Future Work

This paper proposes RADAR-BPO (risk-aware, dynamic, adaptive regulation barrier policy optimization), a novel safe reinforcement learning framework integrating PPO with CBF-based safety filters to mitigate collision risks in mobile robot navigation. The framework leverages PPO for exploratory policy generation while employing the CBF as a real-time safety filter, formulated as a quadratic programming problem, to minimally modify risky actions and ensure collision avoidance. Implemented on a mecanum wheel robot within the ROS Gazebo simulation environment, the method demonstrated significant improvements in safety performance across diverse dynamic and complex scenarios. Comparative experiments against baseline PPO, DQN, and DDPG algorithms confirmed that RADAR-BPO achieves higher success rates, lower collision rates, and superior average rewards while maintaining navigation efficiency, highlighting its effectiveness in balancing exploration with formal safety guarantees.

In future work, the framework could be extended to address more complex real-world challenges. Potential directions include validating the approach on physical robotic platforms to assess its real-time performance and robustness under sensor noise and hardware limitations. Additionally, exploring the use of adaptive or learned CBF parameters to handle heterogeneous obstacle shapes and uncertain dynamics, integrating multi-robot collision avoidance scenarios, and extending the method to unstructured outdoor environments would further enhance this approach’s applicability.

## Figures and Tables

**Table 1:** Trajectory coordinates of pedestrians in environment.

Time (s)	Pedestrian 1		Pedestrian 2	
	x (m)	y (m)	x (m)	y (m)
0.0	0.5	2.5	3.0	0.0
3.0	0.5	1.5	3.0	2.0
3.5	0.5	1.5	3.0	2.0
5.5	0.5	2.5	-	-
6.0	0.5	2.5	-	-
6.5	-	-	3.0	0.0
7.0	-	-	3.0	0.0

**Table 2:** Training parameter settings for RADAR-BPO.

Category	Parameter	Symbol	Value/Range
Policy Optimization	Discount factor	γ	0.99
	GAE parameter	λ	0.95
	Clipping threshold	ϵ	0.15
	Value coefficient	$c_{1}$	0.5
	Entropy coefficient	$c_{2}$	0.01
	Policy iterations	*K*	[150, 200, 300]
	Linear velocity	$v_{\max}$	[0, 0.5]
	Angular velocity	$\omega_{\max}$	[−1.0, 1.0]
Safety Constraints	Safety distance	$r_{\mathrm{safe}}$	0.5
	Max linear velocity	$v_{\max}$	0.5
	Max angular velocity	$\omega_{\max}$	1.0
	Barrier parameter	α	1.0
Robot Kinematics	Half of body’s length	$W_{a}$	0.195
	Half of body’s width	$W_{b}$	0.172
	Characteristic length	$d=W_{a}+W_{b}$	0.367
Training Setup	2D lidar angle range	–	[0°, 360°]
	Scanning range	–	[0.08, 10]

**Table 3:** Algorithm performance comparison.

Environment	Algorithm	Success Count	Collision Count	Average Reward	Collision Rate (%)
Env 1	PPO	27	123	357.23	82.00
	RADAR-BPO	**47**	**103**	**421.58**	**68.67**
	DDPG	17	133	13.67	88.67
	DQN	0	150	271.50	100.00
Env 2	PPO	134	66	377.01	33.00
	RADAR-BPO	**164**	**36**	**1070.09**	**18.00**
	DDPG	25	175	58.02	87.50
	DQN	89	111	273.47	55.50
Env 3	PPO	208	92	399.38	30.67
	RADAR-BPO	**268**	**32**	**762.66**	**10.67**
	DDPG	73	227	19.45	75.67
	DQN	96	204	269.22	68.00

## Data Availability

The original contributions presented in this study are included in the article. Further inquiries can be directed to the corresponding authors.

## Abbreviations

The following abbreviations are used in this manuscript:

AI	Artificial intelligence
IoT	Internet of Things
RL	Reinforcement learning
DQN	Deep Q-Network
DDPG	Deep deterministic policy gradient
CBF	Control barrier function
APF	Artificial potential field
MPC	Model predictive control
PPO	Proximal policy optimization
MDP	Markov decision process
GAE	Generalized advantage estimation
RADAR-BPO	Risk-aware, dynamic, adaptive regulation barrier policy optimization
